# Biobanking as a research accelerator: the perspectives of medical students and interns at a saudi university

**DOI:** 10.3389/fgene.2025.1687927

**Published:** 2026-01-07

**Authors:** Leena AbuOmar, Mariam M. Al Eissa, Hani Tamim, Sadeq Abu-Dawas, Ahmed Yaqinuddin, Khaled Alkattan

**Affiliations:** 1 College of Medicine, Alfaisal University, Riyadh, Saudi Arabia; 2 Public Health Lab, Public Health Authority, Riyadh, Saudi Arabia; 3 Research Centre, King Khaled Eye Specialist Hospital (KKESH), Riyadh, Saudi Arabia; 4 Computational Sciences Department at the Centre for Genomic Medicine (CGM), King Faisal Specialist Hospital and Research Center, Riyadh, Saudi Arabia; 5 Department of Internal Medicine, Clinical Research Institute - American University of Beirut Medical Center, Beirut, Lebanon

**Keywords:** biobanking, biobanks, biospecimen, precision medicine, tissue donation

## Abstract

**Background:**

Biobanks play a significant role in the storage of biological samples for medical research, disease diagnosis at an early stage, treatment, and drug development. The research paper evaluates how medical students at Alfaisal University perceive and understand biobanking, as well as their level of ethical awareness. The study is a gap filler in the current literature base, as it reveals information about what Saudi medical students know and believe regarding biobanking.

**Methods:**

The research employed a cross-sectional method where a survey was administered to medical students and interns at Alfaisal University, Saudi Arabia. A self-administered online questionnaire was used to collect the responses. The questionnaire included 30 questions divided into three subgroups: demographic, knowledge of the principle of biobanking, and perception and attitude towards biobanking. The validity of the questionnaire was evaluated in two directions, i.e., an expert review and a pilot test. The latter sample excluded those who were involved in the pilot stage. The sample size consisted of 457 students, with 72.2% being women and 40% being interns.

**Results:**

The mean of the knowledge scores was 3.93 (SD = 1.63), indicating an average knowledge level. 82.7% of the participants stated that biospecimen donation was a noble activity for society and medical research. 77.7% of the participants advocated for increased resource allocation to biobanking. The dangers of abuse, privacy invasion, and discrimination were brought up, with 81.8% expressing the need for restrictive regulation in medical research. 69.2% of the participants think that a researcher can ensure the interest of the participants, and 4.2% do not believe it is possible. A large proportion of the respondents reported that they would be concerned by the possibility of misuse of the sample (65.2%), confidentiality (64.8%), and discriminatory use (46.2%). Moderate knowledge concerning biobanking (mean = 3.93, SD = 1.63) was observed in the participants, but no significant correlation existed between the knowledge and the desire to contribute biospecimens.

**Conclusion:**

Medical students at Alfaisal University are moderately informed about biobanking and are generally altruistic, although they also express serious ethical concerns about the utilization and safety of their information. Factual knowledge does not necessarily impact the decision to donate, and ethics, trust and positive attitudes should play a significant role in the encouragement of participation in biobanks. These findings highlight the importance of prioritizing open communication and strong ethical safeguards in biobanking ventures, as well as the integration of specialized biobanking education into medical education. This two-pronged approach is necessary to build trust and equip future medical practitioners with the opportunities to facilitate biobanking efforts in Saudi Arabia.

## 1 Introduction and literature review

### 1.1 Overview

Saudi Arabian healthcare has undergone tremendous changes due to technological development, which includes biobanking. Medical practitioners can now preserve and retain delicate body fluids, including nucleic acids, nails, bone marrow, hair, buccal cells, urine, saliva, and blood, for use in treatments and research. Biobanking and precision medicine (PM) are becoming more of interest in promoting patient-specific diagnosis and treatment. Saudi Arabia has achieved several illustrious biobanks that include the Biological Repository Center at King Faisal Specialist Hospital and Research Centre (KFSHRC), the Saudi Biobank at the King Abdullah International Medical Research Center (NGHA), and the National Biobank, which was launched in 2022 to enhance the health of Saudi citizenry by encouraging research and innovation in the prevention of communicable and non-communicable diseases.

### 1.2 Rationale of the study

Despite the increasing number of biobanks across the world, matters pertaining to privacy, increased usage of data over time, and the misuse of genetic information continue to hinder participation. Culture and religion both affect the attitudes of people in Saudi Arabia towards the donation of biological samples. These constitute a significant point to support robust ethical principles, community involvement and the explanation of the rationale and merit of biobanking.

In Saudi Arabia, previous research on awareness of biobanking has been conducted at the population and professional level, but there is also prior research in which various populations were studied at different development stages of biobanks in the country. Al-Ghanim (2009) identified religious factors as a key predictor of attitude towards deceased organ and tissue donation, and one study by Al-Jumah et al. (2011) showed that only a small proportion of the respondents thought that genetic research contradicted religious teachings. According to Merdad et al. (2017), senior healthcare students cited that the most common reasons for not wanting to donate were related to misuse and confidentiality of biospecimens and religious beliefs. More recently, Buhmeida et al. (2022) indicated a gap in the knowledge regarding biobanks and the Human Genome Project among healthcare providers that had an influential role on their willingness to donate biospecimens. This study will investigate medical students at a large private university, which will introduce contemporary-day information regarding the gaps in knowledge, ethical aspects, and new learning requirements.

### 1.3 Aims of the study

The proposed study will examine the existing knowledge of medical students and interns at Alfaisal University in the field of biobanking as a modern research topic and driver. It also examines their willingness to donate biospecimens with the corresponding benefits. The research also addresses ethical concerns related to biospecimen use. The findings will fill an information gap in the existing literature on medical students’ knowledge of biobanks in Saudi Arabia.

## 2 Literature review

### 2.1 Definition of biobanking

Biobanking refers to using, processing, and storing biological samples and relative data in an organized manner of research (Abdelhafiz et al., 2019). Some of the common samples are blood, saliva, urine, buccal cells, bone marrow, and nucleic acids. Linked to demographic and clinical information, these samples may be employed to conduct molecular investigations and studies at the population level. Curation There are large programs like the UK BioBank and the Cancer Genome Atlas (TCGA) that have demonstrated how biospecimen repositories can be curated to facilitate a better understanding of the science of disease and promote precision medicine.

### 2.2 History of biobanks

Contemporary biobanking was associated with the emergence of genetics and biomedical research at the end of the 20th century. The rapid growth became a fact in the 1990s and early 2000s when the research institutions saw the importance of systematically stored biospecimens (Coppola et al., 2019). Such repositories enabled the researcher to carry out longitudinal studies, examine genetic variation, and design specific treatments.

Saudi Arabia has established large institutional biobanks, as a result of which national research capacity has increased, and population-based research has been supported (Buhmeida et al., 2022). Although historical development provides a valuable context, the focus lies in current challenges, e.g., ethical governance, public involvement, and regulatory transparency, which are more relevant to determine the perception of medical students towards biobanking today. These stubborn points merit new studies of their awareness and attitudes in the new environment.

### 2.3 Application of biobanking

Biobanks contribute to genomics, pharmacogenomics, disease mechanism research, biomarkers, and drug development (Simeon-Dubach and Kozlakidis, 2022). Their application to the comparison between affected and healthy individuals assists in determining genetic predisposing factors and defining the proof of disease development (Lazareva et al., 2022). Laboratory Information Management Systems (LIMS) ensure that the traceability of samples and quality of information can be determined over a period.

Measures supported by the biobank data have also been implemented in the precise treatment, finding, and the availability of the population-specific disease patterns (Merdad et al., 2017). They assist the authorities within the field of public health to monitor the trend in diseases and to formulate increased health policies. With the emergence of biobanking in Saudi Arabia, there is growing importance in ensuring that medical students know of its scientific worth, the requirements, and the implications of its ethical aspects on clinical practice.

### 2.4 Types of biobanks

Generally, biobanks can be categorized into three, namely, disease-specific, population-specific, and virtual biobanks. Disease-specific biobanks face limitations regarding the deposition of biological material and data of a given disease, such as cancer, diabetes, or HIV/AIDS. The datasets are disease-specific and disease-focused, and hence, the researchers can analyze them and come up with specific solutions to treatment and management. Population-specific biobanks are those that gather sampling and health information on particular populations or ethnicities, e.g., African Americans, European Americans, or Chinese Americans (Abdelhafiz et al., 2019). These biobanks are used to determine genetic patterns and health trends of specific populations. Biobanks are used during research carried out on the frequency and occurrence of diseases. Digital virtual biobanks are associated with the online management of biological and clinical information. They allow researchers to access, distribute, and collect data remotely. Virtual biobanks also allow an increased number of researchers to use the information from different institutions and geographical regions.

### 2.5 Targeted population

Biobanks are developed to recruit particular target populations in relation to the research objectives. Other biobanks deal with patients who have specific conditions, including cancer, HIV, or diabetes. Others can be easily targeted at specific demographic groups, such as African Americans, in the search for genetic or health-related patterns within that group. Biobanks also collect samples from healthy individuals, which can be used as controls in comparative studies, in addition to those from individuals with illnesses (Baber and Kiehntopf, 2019). The samples aid the researcher in comparing variations between healthy and affected populations and in studying general health patterns among larger populations.

Nevertheless, the sample groups that are commonly included in health research are skewed in many Western nations towards white people who are middle-class and highly educated. It under-represents indigenous groups, minority groups, and individuals of different cultural or language backgrounds. This imbalance is ethically questionable, as it concerns the equitable access and benefit sharing of the research and ultimately renders the research findings unworthy of scientific merit. Lack of representation, particularly across diversity, can introduce inaccuracy in results, which compromises the capacity to establish such inclusive and effective interventions regarding health (Prictor et al., 2018).

### 2.6 Biobanks statistics

Chronic illnesses rely heavily on biobank data in the management and treatment process. They include stroke, Parkinson’s, dementia, cancer, and cardiovascular disease, and in general, they are covered in target populations to undertake research and therapeutics (Conroy et al., 2019). Examples of such data include genetic data, medical records, and patients’ outcomes, such as mortality. It has been observed that publications on biobanks have been on the rise continuously over time. O'Donoghue et al. (2022) state that the total number of studies related to biobanks also increased dramatically, with one article in 1996 and then a high of 747 articles in 2018. On the higher end, Coppola et al. (2019) mentioned that the single year of 2019 generated around 4,061 papers in the biobanks area, which points to an increasing trend.

Depending on the source, there are various estimated numbers of biobanks worldwide. In 2008, the National Association of Biobanks and Biobanking published a national report citing the figures in the University of Biobanking and Biomolecular Resources Research Infrastructure (BBMRI) directory of 641 biobanks in 17 countries. The other report also points out that the majority of biobanks are concentrated in the economically developed states, which means that there is a strong relationship between national income and the investments in biobanking infrastructure. Specifically, in the UK, O'Donoghue et al. (2022) reported 247 biobanks; in Sweden, 116; in the Netherlands, 98; in France, 96; in Germany, 43; and approximately 350 biobanks in Canada. However, the numbers are changeable depending on the database utilized. As an example, the Canadian Tissue Repository Network (CTRNet) and the BBMRI provide various counts of biobanks in Canada. On the other hand, there is a lack of literature concerning the measurement of the number of biobanks in the Middle Eastern countries, meaning that the information on the region is lacking.

### 2.7 Biobanking challenges

Major concerns regarding biobanking are consent and privacy violations. Contribution to a study as a voluntary donor of biological specimens requires informed consent. As a general rule, any person whose body parts or body fluids are sampled to be used in any research should agree to the procedure first with a second party. Informed consent is a moral principle that ensures the rights of participants and protects the autonomy of the subject, particularly children (Alahmad and Dierickx, 2018). The approach ensures that the production of data and samples for biobanks is based on people’s will and knowledge.

In Saudi Arabia, there is an emphasis on the ethics of informed consent to ensure the privacy of information and uphold the privacy of the donor. Strict ethical guidelines must be followed in studies involving human subjects, according to research ethics legislation. These regulations were based on the notion that the rights of the donors would not be infringed upon and that no irresponsible actions would allow the relevant scientific progress to be achieved (Kaust, 2022).

Biobanking is also connected to social life and directly affects communities. Since most people are unclear about the goals, advantages, and societal effects of biobanking, public perception continues to be a significant obstacle. Poor participation is common in this area due to the lack of knowledge, even in the target groups that may offer valuable samples and data to the researchers (Luna Puerta et al., 2020). Mistrust is bred by inadequate information disclosure, which makes people reluctant to participate out of concern about data misuse. In some Muslim communities, there exists a reserve with respect to the biological sample handling procedure, especially when the work is for non-Muslim companies (Khatib et al., 2021). Such skepticism is a problematic issue, revealing the significance of life and judgment, cultural responsiveness, open communication, and acquiring support and trust among the community.

## 3 Materials and methodology

### 3.1 Study design

This study utilized a descriptive cross-sectional type of study, which involved medical students of all academic years at Alfaisal University in Riyadh, Saudi Arabia. The test subjects were all the medical students, both first-year and final-year students. Six questions that were rated as one point each formed the basis of assessing the level of knowledge on biobanking.

### 3.2 Questionnaire validity

The questionnaire was designed and self-administered based on one of the previously validated tools reported in the published article, “Assessment of knowledge about biobanking among healthcare students and their willingness to donate biospecimens” (Merdad L et al., 2017). After requesting permission via email from the original authors, we verified and modified the tool for this particular study. The questionnaire comprised 30 questions, and they were categorized into three parts. An expert panel of public health and research specialists was assembled by Alfaisal University to assess the review and suggest changes. This expert review was followed by a pilot test of the instrument using a sample of 20 students to establish the overall instrument validity. The validation was done in two steps.

To come up with a wide-ranging questionnaire that covered all areas of interest, the knowledge of experts was applied during this phase. The second was to have proper feedback from the pilot respondents on what ambiguity or gap required amelioration prior to the actual implementation of the questionnaire. It is noteworthy that those who were contacted during the pilot research were not given a chance to participate in this study.

The level of knowledge presented on an adapted scale had six knowledge items that were graded 1 (correctly answered) and 0 (incorrectly answered) to give a potential score of 0–6. Some of the sample items consisted of the student’s knowledge of what a biobank is, the purpose of a biobank, and the type of biospecimens that are stored. The items on perception and attitude were measured using a 5-point Likert scale on agreement, neutral, and disagreement to show their consent to the statements indicating ethical concerns, desire to donate, and trust in biobanking practices.

### 3.3 Data collection

The first section consisted of demographics of the participants, such as age, sex, nationality, academic year, and medical history. The second section assessed the perceptions and the knowledge of the participants on biobanking. On the other hand, the third gave them the opportunity to express their thoughts and feelings regarding biobanking as a means of facilitating biomedical research. The questionnaire was developed in English using the Google Forms service. The data collection process took place between March and September 2023, when the online questionnaire link was sent to students via Alfaisal University’s official email system.

The convenience sampling strategy was adopted. The survey link was distributed to all medical students currently enrolled in the university through the official school email system. There were 457 students who responded to take part in the survey. The response rate was estimated at 25% based on the total number of medical students to be used in the data collection period used. The proportion of respondents who were first-to third-year students amounted to 30%, and their experience with research ethics and understanding of biomedical research is a significant drawback when discussing knowledge-related findings.

### 3.4 Data analysis and management

SPSS version 27 was used in data analysis. The categorical variables included gender, nationality, and level of academia, for which descriptive statistics (frequency and percentage) were applied to the measured variables. The normally distributed variables, such as knowledge score and age, were reported as standard deviations and means. The Shapiro-Wilk test was used to assess the normality of the data, and the result indicated a non-normal distribution (p < 0.05).

The Mann-Whitney U test was also used when making comparisons between two independent groups in terms of binary categorical variables. Expected effects for the Kruskal–Wallis H test were assessed using more than two categories of different groups, as in the case of academic level and health background. Spearman’s rank-order correlation was used to analyze the correspondences between ordinal data and continuous variables, such as age, knowledge, perception, and attitude scores. The test used a p < 0.05 significance level.

The knowledge level on biobanking was assessed using six questions, each with one point assigned for a correct answer. Accordingly, there were six correct answers, and zero incorrect answers resulted in the individual score scale (0–6). A 5-point Likert scale was used to measure opinions and perceptions regarding biobanking (1 being strongly disagree and 5 being strongly agree). Middle options were disagreed (2), neutral (3), and agreed (4). Some items were reverse-scored to reveal a pattern in data interpretation, particularly when answering negative statements. For the analysis of attitudes and perceptions, each response on the 5-point Likert scale was split into three groups. The “Agree” and “Strongly Agree” answers were counted as “Agreement.” “Disagree” and “Strongly Disagree” answers were counted as “Disagreement.” The analysis left “Neutral” on its own and studied it apart from the other two groups.

The Cronbach’s alpha was used to test internal consistency of the perception and the attitude scales. The scales of perception and attitude were associated with acceptable and good reliability, respectively. The knowledge scale was constantly rated out of 0–6 based on the research to maintain consistency in scoring. This sampling of the respondents implies that some respondents had a low amount of previous exposure to research ethics or biomedical research, and the scores in the knowledge should be viewed with such consideration.

### 3.5 Ethical considerations

The ethics plan of the study received positive ethics approval on 18 December 2022, reference number IRB-20194, by the Institutional Review Board (IRB) of Alfaisal University. The questionnaire was completed, which presupposed the informed consent and participation in the study were purely on a voluntary basis. To maintain privacy of the respondents, all the answers were anonymized, the research team kept full control of the data, and it can only be utilized in academic research. The presence of encrypted information digitally increased the security and confidentiality of data.

## 4 Results

### 4.1 Demographic characteristics of participants

**FIGURE 1:**
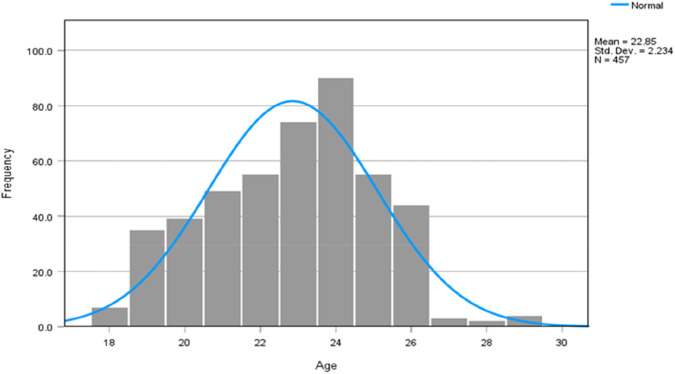
Sample distribution by age.

Based on the demographic profile of the study participants, most of the sample consisted of women (72.2%), and the remaining 27.8% were men. The mean age of the participants was 22.85 years, with a standard deviation of 2.234 (Figure 1). The fact that the proportion of non-Saudis present is a bigger percentage (regarding the nationality) indicates that younger adults are predominant in the sample. Based on the distribution of education, no measurable differences concerning the academic status of the participants in the College of Medicine were found. The rest of the members were spread through the years of the medical school, with a total of 17.1% consisting of the fifth-year students (Table 1).

**TABLE 1:** Demographic composition of study participants.

Attribute		Frequency
Gender	Female	330 (72.2%)
	Male	127 (27.8%)
Nationality	Non-Saudi	308 (67.4%)
	Saudi	149 (32.6%)
The current academic level at your college of medicine	1st year	45 (9.8%)
	2 nd year	41 (9.0%)
	3rd year	53 (11.6%)
	4th year	57 (12.5%)
	5th year	78 (17.1%)
	Internship	183 (40.0%)
How would you rate your general health status?	Very good	199 (43.5%)
	Excellent	151 (33.0%)
	Good	92 (20.1%)
	Fair	12 (2.6%)
	Poor	3 (0.7%)
Has a doctor or any health professional ever diagnosed you with a chronic disease (e.g., heart disease, diabetes, stroke, cancer, etc.)?	No	406 (88.8%)
	Yes	51 (11.2%)
Is there a history of inherited disease in your family?	No	323 (70.7%)
	Yes	134 (29.3%)
Have you ever had a blood test?	No	27 (5.9%)
	Yes	430 (94.1%)
Have you ever donated blood?	No	303 (66.3%)
	Yes	154 (33.7%)
Have you ever had a tissue test (e.g., biopsy, buccal swab, etc.)?	No	358 (78.3%)
	Yes	99 (21.7%)
Have you ever donated tissue/an organ?	No	452 (98.9%)
	Yes	5 (1.1%)
Have you ever been a participant in health-related research?	No	203 (44.4%)
	Yes	254 (55.6%)
Age (mean ± S.D)		22.85 ± 2.234

The past medical history of the respondents was also determined against chronic diseases. The overwhelming majority (88.8%) had never had a professional health worker diagnose them with a chronic illness. Meanwhile, 11.2% reported a diagnosis of a disease such as cancer, diabetes, stroke, or cardiovascular disease. Concerning hereditary health issues, 70.7% of the respondents reported no known family history of inherited diseases, indicating a low prevalence of hereditary issues among the participants (see Table 1).

In the meantime, 29.3% of the respondents reported that they grew up in families with a history of genetic disorders. This data provides information on the background of the participants regarding a genetically related health issue. Furthermore, the results of medical testing and participation in studies show that a significant number had already experienced blood tests (94.1%) and tissue tests (21.7%). Fewer, however, reported a history of participation in blood (33.7%), tissue or organ (1.1%), or health-related research studies (55.6%) (see Table 1).

A histogram showing an age-wise distribution of the participants is given in Figure 1 has a slightly right-skewed trend. The largest bars are found in the 18- to 25-year-old age category, with the highest concentration at ages 23 and 24. The rates of participants decline visibly with further advancing age, creating a tapering tail to the right. This trend indicates that the sample is largely comprised of younger adults between the ages of 18 and 30 years.

### 4.2 Biobanking knowledge

A set of specific questions was used to assess the participants’ knowledge of biobanking. Regarding the term “biobank,” 44.9% of respondents (205 respondents) indicated that they were familiar with it. When probed about its principal operation, 63.7 percent (291 people) identified the correct purpose, which is the retrieval and stratification of biospecimens used in diagnostics and biomedical studies. 42.0% (n = 192) of participants correctly defined biospecimens in modern biobanking as biological samples and related biomolecules, along with correlated lifestyle, socioeconomic, and clinical data. Additionally, a sizable majority (79.9%) concurred that anonymity and confidentiality should be maintained for data from annotated biospecimens. In addition, 89.3 percent of the respondents believed that hidden consent is a must-have preceding the donation of biospecimens. Also, 73.3% (335 participants) admitted that they had standardized procedures under which biobanks collect, treat, store, and distribute biospecimens. The total knowledge score had a mean of 3.93, with a standard deviation of ±1.63, calculated by assigning one point to each correct answer (see Table 2).

**TABLE 2:** Participants’ biobanking knowledge.

Attribute		Frequency
Have you ever heard of the term biobank?	No	252 (55.1%)
	Yes	205 (44.9%)
What do you think the purpose of a “biobank” is?	Collect and store biospecimens for diagnostic and treatment purposes only	32 (7%)
	Collect and store biospecimens for research purposes only	38 (8.3%)
	Collect and store biospecimens for diagnostic, treatment, and biomedical research purposes	291 (63.7%)
	Do not know	96 (21%)
According to modern biobanking, biospecimens mean	Samples and/or biomolecules only	26 (5.7%)
	Samples and/or biomolecules with annotated clinical data only	54 (11.8%)
	Samples and/or biomolecules with annotated clinical, socioeconomic, and lifestyle data	192 (42%)
	Annotated clinical, socioeconomic, and lifestyle data only	31 (6.8%)
	Do not know	154 (33.7%)
Do you think biospecimen annotated data (i.e., related data) should be confidential and anonymous?	Yes	365 (79.9%)
	No	18 (3.9%)
	Do not know	74 (16.2%)
Do you think donating a biospecimen to a biobank requires signing a consent form?	Yes	408 (89.3%)
	No	6 (1.3%)
	Do not know	43 (9.4%)
Do you think that there is a standard operating procedure (SOPs) for biobanks to collect, process, store, and release biospecimens?	Yes	335 (73.3%)
	No	11 (2.41%)
	Do not know	111 (24.3%)
Biobanking knowledge		3.93 ± 1.63

Even though, on the overall trend, there is a trend of familiarity with various foundational concepts, the average score of 3.93 out of 6 manifestations means a moderate but not a high level of knowledge and cannot be taken to mean that students are well informed regarding biobanking.

**FIGURE 2:**
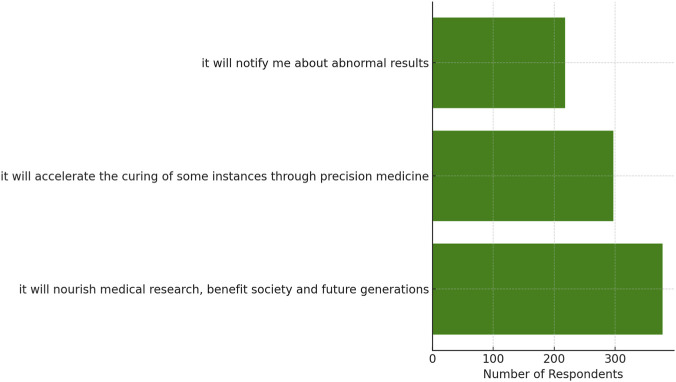
Perceived benefits of biospecimen donation.

Table 3 describes the survey of participants and the reasons they provided for donating biospecimens to a biobank. A considerable proportion (just above 82.7%) of participants (n = 378) thought that their donation would help society develop and leave future generations better off by facilitating medical research (Figure 2). Moreover, 65% of respondents noted the contribution of biospecimen donations to precision medicine, which could accelerate the treatment process for a particular disease. At the same time, 47.7% stated that the donation of biospecimens might be an opportunity to identify some abnormal outcomes and obtain possible personal health information.

**TABLE 3:** Benefits and drawbacks of biospecimens donation in modern biobanking.

Attribute		Frequency
According to modern biobanking, what are the benefits of biospecimen donation?	It will nourish medical research, benefit society, and future generations	378 (82.7%)
	It will accelerate the curing of some instances through precision medicine	297 (65.0%)
	It will notify me about abnormal results	218 (47.7%)
According to modern biobanking, what are the drawbacks of biospecimen donation?	Concern about the misuse of biospecimens in biomedical research	298 (65.2%)
	Concern about confidentiality and privacy breaches	296 (64.8%)
	Concern that biological or personal information may be used for discriminatory purposes	211 (46.2%)
	Fear of pain associated with biospecimen donations, such as injections and needle pricks	188 (41.1%)
	Religious reasons	151 (33.0%)

**FIGURE 3:**
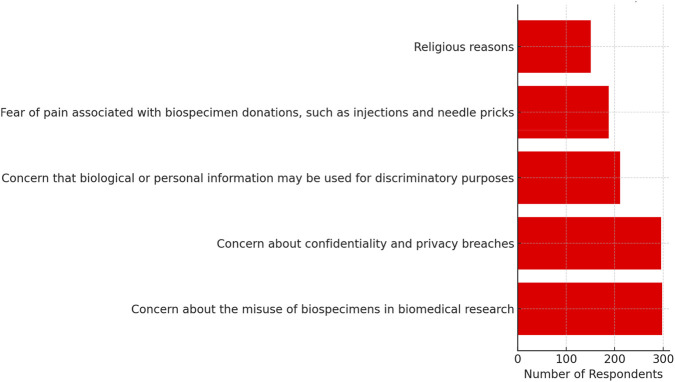
Perceived drawbacks of biospecimen donation.

The respondents were asked to express their concerns about the potential negatives associated with providing biospecimens in modern biobanking, as shown in Table 3. These results indicate that respondents expressed a diversity of issues. The possibility of biospecimens being misused in biomedical studies was the main concern of the largest percentage, 65.2% (n = 298) (Figure 3). Violation of privacy and confidentiality came next, with 64.8% of respondents reporting on the issue. Furthermore, the respondents indicated worry over the subject of discrimination given the possibility of using their personal/biological information, with 46.2% of the respondents indicating worries in this field. The presence of physical discomfort, which includes pain during needle pricks and injections, was experienced by 41.1% of those who responded. Lastly, only a third of them mentioned that religious beliefs may serve as an obstacle to biospecimen donation.

### 4.3 Participants’ perception and attitudes towards biobanking

Participants’ attitudes toward the practice of biobanking in relation to the general field of medical research revealed a wide range of perceptions. The majority of the 457 medical students polled expressed a positive outlook. Specifically, 96 participants (21.0%) expressed strong agreement with the statement that biobanking is a valuable contribution to medical research, while 208 participants (45.5%) agreed. Conversely, only a small percentage disagreed, with 3 (0.7%) strongly disagreeing and 10 (2.2%) disagreeing. Although there is still a lot of reluctance, the public’s willingness to donate biospecimens indicates a significant opportunity. Although 50.3% of participants said they would be willing to donate, with 35.2% agreeing and 15.1% strongly agreeing, this favorable opinion is offset by the 33.8% who previously disagreed. This division draws attention to a crucial knowledge and trust gap that biobanking efforts need to fill. Conversely, there was some hesitancy, as 45 respondents (9.8%) and 10 respondents (2.2%) strongly disagreed with donating their biospecimens. Many of them disagreed that medical researchers use personal gain as motivation, with 156 participants (34.1%) disputing that statement and 48 (10.5%) arguing strongly against it. On the other hand, 85 people (18.6%) agreed with this point, whereas 31 people (6.8%) strongly agreed (see Table 4).

**TABLE 4:** Participants’ attitude towards biobanking.

Attribute		Frequency
I Have a positive view of biobanking in medical research	Strongly disagree	3 (0.7%)
	Disagree	10 (2.2%)
	Neutral	140 (30.6%)
	Agree	208 (45.5%)
	Strongly agree	96 (21.0%)
I Will donate a biospecimen (e.g., saliva/sputum, urine, blood, buccal swabs, toenails, hair, my excess surgical tissue, a deceased family member’s organs or tissues, etc.) to a biobank to perform biomedical research	Strongly disagree	10 (2.2%)
	Disagree	45 (9.8%)
	Neutral	172 (37.6%)
	Agree	161 (35.2%)
	Strongly agree	69 (15.1%)
Medical researchers are mainly motivated by personal gain	Strongly disagree	48 (10.5%)
	Disagree	156 (34.1%)
	Neutral	137 (30.0%)
	Agree	85 (18.6%)
	Strongly agree	31 (6.8%)
Medical researchers can be trusted to protect the interests of people who take part in their research	Strongly disagree	5 (1.1%)
	Disagree	14 (3.1%)
	Neutral	122 (26.7%)
	Agree	222 (48.6%)
	Strongly agree	94 (20.6%)
Medical researchers can influence others by volunteering for medical research	Strongly disagree	4 (0.9%)
	Disagree	11 (2.4%)
	Neutra	101 (22.1%)
	Agree	233 (51.0%)
	Strongly agree	108 (23.6%)
Participating in medical research is generally safe	Strongly disagree	8 (1.8%)
	Disagree	29 (6.3%)
	Neutral	146 (31.9%)
	Agree	205 (44.9%)
	Strongly agree	69 (15.1%)
Modern science poses more harm than good	Strongly disagree	36 (7.9%)
	Disagree	118 (25.8%)
	Neutral	152 (33.3%)
	Agree	107 (23.4%)
	Strongly agree	44 (9.6%)
Society needs to devote more resources to medical research	Strongly disagree	3 (0.7%)
	Disagree	2 (0.4%)
	Neutral	97 (21.2%)
	Agree	226 (49.5%)
	Strongly agree	129 (28.2%)
Medical research needs to be closely regulated in order to prevent harm to research participants	Strongly disagree	6 (1.3%)
	Disagree	2 (0.4%)
	Neutral	75 (16.4%)
	Agree	193 (42.2%)
	Strongly agree	181 (39.6%)
Medical research will find cures for many major diseases during my lifetime	Strongly disagree	4 (0.9%)
	Disagree	7 (1.5%)
	Neutral	113 (24.7%)
	Agree	192 (42.0%)
	Strongly agree	141 (30.9%)

The measurement of the degree to which people trust that medical researchers will protect the interests of participants was highly encouraging. Almost half of the participants (222; 48.6%) agreed that they could trust the researchers, and a good 94 (20.6%) participants strongly agreed. A minority of 14 (3.1%) disagreed, and 5 (1.1%) strongly disagreed. In a question on whether medical researchers motivate others by voluntarily taking part in research, 51 percent (233 people, 51.0) responded that they agree, and 23.6 percent (108 people) strongly agree. Very few respondents disagreed, with 11 (2.4%) disagreeing and 4 (0.9%) strongly disagreeing. A total of 101 participants (22.1%) expressed no opinion (see Table 4).

The attitudes towards the safety of participation in medical research were also positive. Out of 457 respondents, 205 (44.9%) agreed that it is safe to participate, and 69 (15.1%) strongly agreed. Nevertheless, 29 (6.3%) did not agree, and 8 (1.8%) strongly disagreed. One hundred and forty-six (31.9%) respondents had a neutral position (see Table 4).

There were even more split views on whether modern science and technology bring more harm than good. There was a stark difference in the opinions: 33.8% (n = 154) disagreed (25.8% strongly disagreed, 7.9% disagreed), while an almost equal 33.3% (n = 151) agreed. The other 152 respondents (33.3%) assumed an undecided stand (see Table 4). Most people agreed on the need to allocate more resources towards medical research. Out of the total number of 455 responses, 226 participants (49.5%) agreed, and 129 (28.2%) strongly agreed. The numbers that disagreed with it were minimal, with only two (0.4%) disagreeing and three (0.7%) strongly disagreeing. Ninety-seven (21.2%) participants took a neutral stance (see Table 4).

The respondents also exchanged opinions regarding the control of medical research. There was a high level of agreement, with 193 (42.2%) agreeing that strict oversight is essential to safeguard participants and 181 (39.6%) strongly agreeing. Eight (1.7%) people disagreed with this perspective, with two (0.4%) disagreeing and the other six (1.3%) strongly disagreeing. One hundred and sixty-four (33.9%) were neutral (see Table 4).

Lastly, the perspective on the future of medical research presented a hopeful outlook. Forty-two percent of respondents agreed that this is true, with 30.9 percent strongly agreeing that research will yield many cures for major diseases in their lifetime. Only 1.5% of the respondents disagreed, and 0.9% strongly disagreed, with 24.7% staying neutral (see Table 4).

OLS regression was used to determine the predictors of biobanking knowledge in the participants. Positive predictors of knowledge were age and an opinion that medical research would achieve cures in their lifetime (p < 0.01). Other variables like gender, nationality, overall health, blood or tissue donation and tastes towards biobanking were not significant. In total, the model explained 27.1% variance in the scores of knowledge (Adjusted R2 = 0.187). These findings explain that the age of the student and optimism in medical research are the key variables that have weak statistical associations with knowledge of biobanking.

The association involving knowledge and readiness to give biospecimens was also determined using OLS regression. The level of knowledge did not significantly predict willingness to donate (p = 0.217). Age had a weak but significant positive impact (p = 0.046), which was not significant in the case of gender, nationality and health status. The model was only able to explain 2.5% of the variance (R2 = 0.025). These findings suggest that though the knowledge of the student had a slight age difference, it did not directly influence the desire of the individual to donate to the recipients. They imply that there are other attitudinal or motivational determinants of the decision to donate.

### 4.4 Correlation analysis

Table 5 presents the means and standard deviations of knowledge ranked by demographic sections regarding biobanking awareness. The data is classified according to four main demographic elements: age, academic level, nationality, and gender. Among the age groups, 24-year-old individuals had the highest average knowledge score (1.31 ± 0.32), indicating a better understanding of biobanking concepts among individuals in the same age category.

**TABLE 5:** Biobanking knowledge characteristics by demographic variables.

Variables	Categories	Biobanking knowledge		*p*-value
		Average knowledge	Standard deviation	
Age	18–21	1.00	0.51	<0.001
	22–25	1.28	0.38	
	26–29	1.29	0.39	
Academic level	1st year	0.90	0.57	<0.001
	2nd year	0.97	0.53	
	3rd year	1.09	0.44	
	4th year	1.30	0.30	
	5th year	1.30	0.34	
	Internship	1.29	0.40	
Nationality	Non-Saudi	1.21	0.43	<0.001
	Saudi	1.19	0.45	
Gender	Female	1.20	0.45	0.976
	Male	1.21	0.41	

**FIGURE 4:**
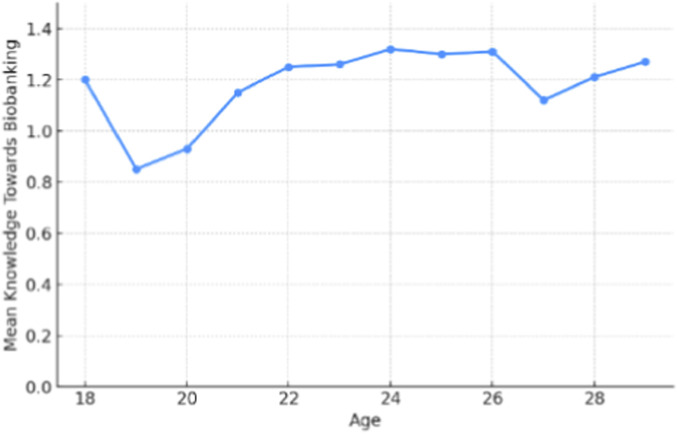
Mean knowledge average towards biobanking by age.

**FIGURE 5:**
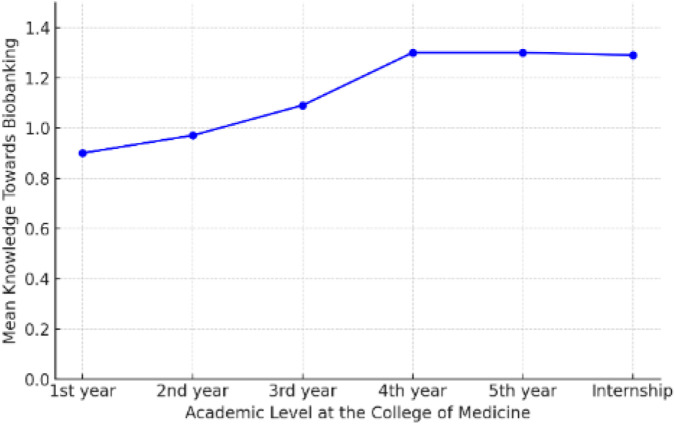
Mean knowledge average towards biobanking by academic level.

Conversely, younger participants aged 19 had the least mean knowledge of biobanking (0.84 ± 0.61), which is quite low compared to their elder peers (Figure 4). The observed knowledge gap between age cohorts is probably explained by differences in educational access, attainment, and personal interests. In terms of academic standing, students in their fourth and fifth years of study had the highest average knowledge level scores (1.30 ± 0.30 and 1.30 ± 0.34, respectively). This pattern suggests that by taking advanced coursework or developing a closely related practical interest in the field, one may be able to learn more about biobanking. The first year had the lowest mean knowledge score (0.90 ± 0.57), suggesting that those just starting their academic careers may not know enough about biobanking (Figure 5).

**FIGURE 6:**
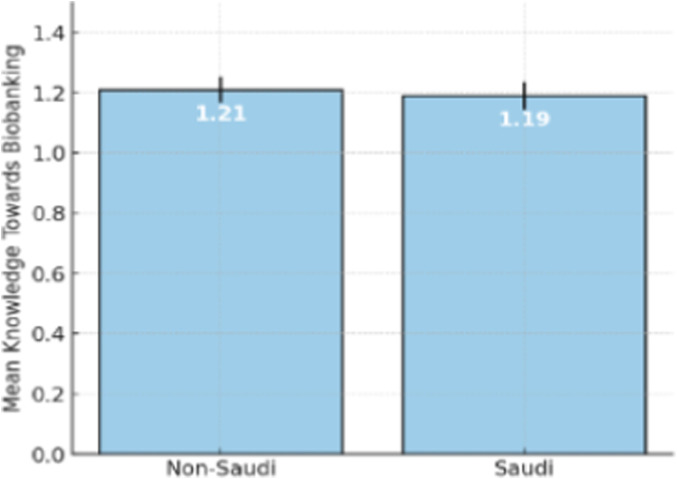
Mean knowledge average towards biobanking by nationality.

Evaluation of the nationality of respondents showed a relatively small variance in the mean knowledge score between Saudi and non-Saudi people. The mean score of non-Saudi participants (1.21 ± 0.43) was slightly higher than that of Saudis (1.19 ± 0.45) (Figure 6). The gap is largely insignificant, but it could be an indication of a small difference in one or more of the following: exposure to education in biobanking, cultural outlook, or the reach of available information on biobanking between the two populations.

**FIGURE 7:**
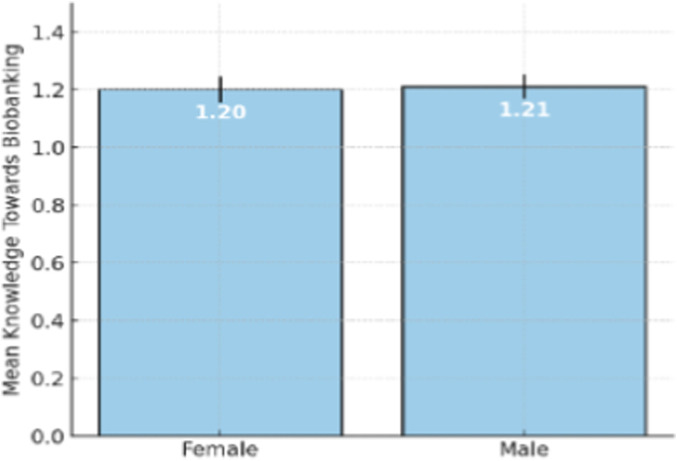
Mean knowledge average towards biobanking by gender.

The measured level of difference between men and women in terms of knowledge regarding biobanking revealed a small disparity in the results, with males having an insignificantly elevated mean (1.21 ± 0.41) compared to women (1.20 ± 0.45). Such a slight difference suggests that gender may not significantly influence the interpretation of biobanking concepts among participants in the sampled population (Figure 7). The impending insignificance may be the result of personal interests, dissimilarity in the degree of academic experience, or varying degrees of commitment to biobanking-related materials rather than gender-related variables.

## 5 Discussion

### 5.1 Summary

The aim of the study was to determine knowledge, perception, and attitudes of medical students towards biobanking and to establish some important findings. The extent of factual knowledge was moderate, the perception of biospecimen donation value was mostly positive, and the strength of altruism as a motivation to become a biospecimen donor was high. Simultaneously, concerns regarding the privacy, sample abuse, and doubts concerning the intentions of researchers remain. All these outcomes are indicative of positive thinking, as well as the areas that require further education and reassurance.

The foundation of biobanking is voluntary participation, which is an essential component of contemporary medical research. The study assessed Alfaisal University medical students’ level of awareness, perception, and attitudes regarding biobanking. The study collected responses from 457 students at different academic levels using a cross-sectional survey to gauge their familiarity with biobanking concepts and associated ethical concerns as well as their willingness to support biomedical research. Most of the respondents were women (72.2%), and the academic background of the sample was diverse, with more than 40.0% of them being in an internship year. Male/female gender, nationality, academic year, general medical status, likelihood of chronic diseases, family health records, prior medical test experience, and history of research participation were among the full range of demographic variables examined. The fundamentals of biobanking were generally well understood. Remarkably, the great majority of respondents accurately identified the primary function of biobanks as collecting and preserving biological samples for use in scientific research, drug development, and diagnostics.

Upon study evaluation, participants showed a rather medium level of knowledge about biobanking, with an average knowledge score of 3.93 out of 6 (Table 2). The survey also explored the reasons for and concerns about the donation of biospecimens. The anticipated value of such contributions to society and medical advancements was a very strong sentiment volunteered by a good number of respondents. However, a number of participants expressed worries about privacy violations, misuse of biospecimens, and the repercussions of using their personal data in a discriminatory manner for research purposes. These problems brought to light some of the biggest privacy and ethical concerns that other participants had about biological material donation.

Overall, the student attitudes and views in relation to biobanking were largely favorable. It was believed that biobanking had great significance in the medical research establishment. Whilst there were those respondents who did not consent to agree or to adopt a neutral stand on the controversy around biobanking, many of them have been very cooperative in making available their biospecimens to researchers with alleged health and social advantages. However, not all respondents felt entirely supported by the apparent clarity of the intentions that were driving medical research, and this uncertainty apparently affected their readiness to take up such actions. However, the overwhelming majority of them still had faith in the motives of medical researchers because they were sure that the best interests of the participants would be taken into account over the danger of the experiment.

Although the general impression is favorable, the research also demonstrates conflicting attitudes to the integrity and safety of biobanking practices, which attest to a diversity of admirable opinions in the group. These revelations create a great context of how medical students feel about biobanking, including both the support and the moral issues. These results have significant implications for medical education, which necessitates introducing sessions on tobacco banking issues into the academic program, thus building more knowledge and awareness to contribute to the creation of informed participation in the biomedical research environment.

However, there are a number of limitations that should be taken into account. The fact that the research involved a medical student group from one institution, Alfaisal University, limits the external validity of the study to other groups. Furthermore, the information was self-reported, and this may result in the risk of a response bias. Moreover, the research is also cross-sectional and, thus, reflects the perceptions of the participants at a certain point. This study describes the knowledge, attitudes, and perceptions of medical students regarding biobanking. It also highlights how important focused educational initiatives are to fostering ethical giving and establishing trust, both of which are necessary for the effective deployment of biobanking.

### 5.2 Interpretation of results

The emphasis of the discussion is on the interpretation of the findings rather than the repetition of the background ideas developed in the introduction. The interpretation is only guided by the results, which directly came about as a result of the analysis.

#### 5.2.1 Demographic characteristics

The demographic characteristics to be discussed in this segment of the report are the age, gender, and income level of the targeted audience. As Table 1 demonstrates, the demographic composition of participants is somewhat interesting, and its trends can be taken into account. One of the most impressive ones is the high gender ratio: 72.2% of the sample population consists of females and 27.8% of males. This is an area that should be filled in, particularly when it comes to the explanation of how gender-specific variables can affect attitudes and the level of awareness regarding money in biobanking. The average age of the sample was 22.85 years, and it was rather homogeneous, as the sample included rather young medical students. At 67.4%, the non-Saudi nationality sample size was likewise sizable. This indicates that a variety of cultural backgrounds were represented in the population. Potential disparities in attitudes and perceptions regarding biobanking are brought about by this type of multicultural representation.

Participants’ academic backgrounds varied, with the majority (40.0%) being in their internship year. The debate concerning the value of academic experience, which should be viewed as something that shapes one’s conceptual knowledge and the vision of biobanking concepts, is sparked by this kind of variation in the evolution of educational levels. Additionally, the respondents had a low prevalence of chronic diseases and were in good health. These health determinants are also important because they may influence how receptive the respondents are to ideas like organ donation and sample contribution to medical research.

Recently, several intriguing demographic trends have emerged that merit further investigation. This high percentage of female respondents is typical worldwide, as more and more women are pursuing degrees in medicine and other healthcare-related fields (Merdad et al., 2017). Likewise, the presence of a significant number of non-Saudi members demonstrates the global composition of the student body, which also implies that cultural diversities could subtly affect biobanking awareness and engagement (Prictor et al., 2018). Proper insight into such demographic distribution helps in the greater analysis and helps in the attempt to make more inclusive and responsive promotions of participation in biobanking.

It was revealed that there is a statistically significant, though tiny, positive correlation between knowledge about biobanking and age. This implies that the effect of increasing familiarity with the concepts of biobanking as students age is consistent with past research findings that are in line with the studies that equate health-related knowledge domain proficiency with age (Malsagova et al., 2020). However, since the strength of this association was rather low, age might not be a determining factor in defining the difference in biobanking awareness in a medical student population (Lazareva et al., 2022). Other reasons have a more significant role.

Notably, there was no significant variation in the aspects of biobanking knowledge depending on nationality or gender. The result compares with previous results that found knowledge and attitude gaps associated with cultural and gender-based factors (Alahmad and Dierickx, 2018). The non-existence of such variations in this work pinpoints the urgency of individualized educational programs guaranteeing equal availability of biobanking information to all student groups. Medical institutions can close informational gaps and create a more inclusive learning environment to better prepare future healthcare professionals to participate and contribute to the development of biobanking and biomedical research, irrespective of cultural or gender background (Coppola et al., 2019).

#### 5.2.2 Biobanking knowledge

This study evaluated the respondents’ knowledge of biobanking based on the responses in Table 2, providing an insightful look at their attitudes. The percentage of respondents who had heard the term “biobank” before, which was 44.9%, was used to gauge awareness. According to the findings, biobanking is a stratified conceptualization, meaning that the majority of respondents understand the basics of the concept. Most interestingly, there were only 63.7 percent of those who responded accordingly by saying that the purpose of biological specimen storage and collection in biobanks is to use them in diagnosis, treatment, and research. This is a positive trend with the results presented by Merdad et al. (2017), since only 27% of the students knew the term, which indicates the spreading awareness about the concept of biobanking.

The target population had a moderate level of understanding, as indicated by the mean general knowledge score of 3.2 out of a possible 7 (SD = 0.6). This is a central tendency and dispersion measure indicator, which points to an ever-growing yet still-developing appreciation of biobanking with respect to its role in medical science. Secondly, the survey revealed that people had varied levels of awareness about the types of biospecimens, with 42.0 percent of the respondents providing the correct responses to the exact categories of samples related to biobank practices. Moreover, the participants also pointed out ethical and legal issues surrounding biospecimen collection and storage. They were particularly keen on giving consent and privacy to the donors.

The resultant average knowledge score was 3.93 with a standard deviation of 1.63, which shows that the sample possessed a positive level of knowledge. Such statistics demonstrate that the role of biobanking in medical science is becoming more and more known, however immature. There was also a difference in familiarity with biospecimen types; 42.0% responded well and identified the specific sample types involved in biobank activities. Also involved, participants were aware of the legal and ethical concerns with regard to consent and privacy of the donor.

The sample size of 20 accepts the general good and positive knowledge level, with an average of 3.93, having a standard deviation of 1.63. These discoveries signify improvement, but they show an area of education and awareness weaknesses. As a result, the use of systematic programs is crucial to increase biobanking literacy. These findings can be incorporated into more general debates about biobank education and the need to introduce specific interventions to increase the basic understanding of this very important field of medical research.

#### 5.2.3 Motivations and concerns

Motives to give biospecimens were examined, and altruism surfaced as the most influential feeling among people. Table 3 indicates that 82.7% of them stated that they believed that the donation of biospecimens would have a beneficial effect on society and future generations. It is characteristic of a high rate of civic self-service and an interest in the development of medical science. The majority of the participants consider biospecimen donation as an important input towards human health and scientific advancement. Three-quarters of them are of the view that donations have the potential to hasten the production of customized cure agents for specific infections (Table 3). Such an inclination to precision medicine demonstrates that the donors cherish the knowledge of a treatment specific to the patient profile, and donation is a selfless behavior that the present and future health systems have a long-term consequence for. Also, in tandem with optimism, the study presented the issue of misuse of biospecimens. The greatest concern was scientific exploitation, where 65.2 percent of people indicated their concern (Table 4). This consciousness indicates moral concerns and the readiness to take into account the laws that do not allow any abuse. Moreover, 64.8 percent were afraid of a privacy breach, and 46.2 percent were worried that their genetic or personal information was going to be used to discriminate against them (Table 4). These results draw attention to important moral issues with the governance and donation process. We must put strong ethical standards, open communication, and extensive educational campaigns into place in order to allay these worries and win over donors. Such findings concur with previous works on the ethical aspects of human biobanking. One systematic review performed by Budimir et al. (2011) on 145 studies determined that as the field of human biobanking grows and advances, so will the accompanying ethical dilemmas regarding it. Hence, continuous thought and discussion are indispensable. Therefore, the present study presents useful information on issues and values that motivate the populace to provide biospecimens and can be used to make future data collection projects ethically acceptable and practical.

#### 5.2.4 Perceptions and attitudes towards biobanking

According to the findings as indicated in Table 4, the majority of participants expressed a positive view toward biobanking. Biobanking has a positive impact on medical research, according to 66.5% of respondents overall, with 45.5% agreeing and 21.0% strongly agreeing. The findings imply an overall recognition of its usefulness in the growth of science and the enhancement of health outcomes. The mixed answers, however, appeared when it came to the aspect of personal willingness to donate the biospecimens. It is important to note that 35.2% and 15.1% of those who participated in the study agreed and strongly agreed to provide their biological samples; however, 12% vehemently rejected or strongly rejected. This indicates that some participants still have reservations, which may be due to ethical or privacy concerns.

The intentions of researchers were also checked. Approximately 34.1 percent of respondents did not approve of researchers who had self-interest as their primary motivator. This depicts that most people have trusted the good faith of the scientific community. Only 4.2% of participants do not think a researcher can protect their interests, compared to 69.2% who do. The findings indicate how transparency and ethical conduct are vital in developing trust.

Most participants thought medical research was safe; 60.0% of them thought there was little risk of involvement (44.9% strongly agreed, 15.1% agreed). But the question of whether modern science is more harmful than beneficial is even more disputable. A third (33.3%) think that science is dangerous, and they question the ethical and social aspects of working in science. There was a strong backing for increased funding. 66.7 percent of those interviewed indicated that there was a need to allocate more money to scientific development. Similarly, 81.8% responded to the question that medical research should be highly subject to control to avoid the negative impact. This indicates significant consciousness of ethical requirements and rigid regulation.

Generally, the respondents were optimistic towards biobanking. The majority of people think that it is a worthy practice in high-level medical research. The respondents regarded biobanking as a good thing for society and generations to come, on the basis of altruism (Alahmad and Dierickx, 2018). Such a self-sacrificial attitude can be compared to the concept of biological citizenship, according to which biobanking is perceived as an activity that contributes to the common good and scientific development (Malsagova et al., 2020). Speaking of the positive impressions, it should be mentioned that individuals should be educated and interested in the work of biobanks to minimize insecurities, as well as motivate them to take part.

Nevertheless, the risks associated with the donation of biospecimens were also an issue of concern. The respondents raised the issue of inappropriate use, lack of confidentiality, and mismanagement of samples during research. These concerns demonstrate how useful ethical standards and governmental regulation of the donors can be in helping enhance transparency (Coppola et al., 2019). The biobank programs are supposed to emphasize community and transparency to build trust between the institutions and the general population. Stakeholders’ anxieties can be allayed and their involvement in the decision-making process increased by educating them (Lazareva et al., 2022).

Even though the level of knowledge regarding biobanking was moderate (mean score 3.93 ± 1.63), it did not correlate with willingness to donate biospecimens significantly (OLS regression, β = 0.052, p = 0.217, R2 = 0.025) (Table 6). The positive effect of age was very weak (β = 0.041, p = 0.046), but significant, and the variables gender, country of origin, and health status were not significant predictors. Those findings suggest that factual knowledge alone does not serve as motivation to take part. The attitudes, the confidence in the researchers, the ethical factors and the selfless intentions appear to be more defining. As a result, the intervention to improve the donation rates must entail education and specific interventions to establish positive perceptions of taking part in the research and working to overcome the ethical concerns.

**TABLE 6:** OLS regression predicting willingness to donate biospecimens.

Predictor	Coefficient (β)	Std. Error	t-value	p-value	95% CI lower	95% CI upper
Constant	2.231	0.480	4.647	0.000	1.288	3.175
Knowledge score	0.052	0.042	1.237	0.217	−0.031	0.135
Age	0.041	0.020	2.002	0.046	0.001	0.080
Gender	−0.113	0.098	−1.153	0.250	−0.305	0.079
Nationality	−0.035	0.093	−0.380	0.704	−0.218	0.148
Health status	0.079	0.053	1.492	0.137	−0.025	0.183

Model statistics: *R* ^2^ = 0.025, Adjusted *R* ^2^ = 0.014, F (5,451) = 2.272, p = 0.046.

#### 5.2.5 Implications for medical education and research

The results showed that participants were generally optimistic about the direction of medical research. The percentage of people who agreed or very much agreed with the statement that major diseases would be cured during their lifetime was 42.0 and 30.9, respectively (Table 4). The best illustration of such optimism is a shared idea of the constant development of medical science. This positive thinking shows the great confidence that individuals place in the possibility of future research to bring about major discoveries. There seems to be the belief among respondents that, eventually, scientific breakthroughs will provide the much-needed changes in medical care. This non-individualized optimism has the potential to become an incentive for making more people become engaged and invest in the research. Stressing the importance of medical advancements can inspire people to carry out and participate in research.

This positive attitude could influence the long-run culture to promote scientific innovation. It is essential to cultivate this viewpoint because it generates the public support required to accomplish significant advancements in healthcare. The study also showed clear disparities in the biobanking knowledge among scholars. These differences highlight the necessity to include biobanking education in the medical school curriculum (Merdad et al., 2017). This is the reason why starting on the themes of biobanking early would help the students to have a more concrete picture of the ethical and scientific aspects of biospecimen research (Prictor et al., 2018).

Knowledge gaps in biobanking can be successfully filled with focused educational interventions that are adapted to the learner’s level. By increasing awareness of its procedures and practices, such training would give aspiring medical professionals the in-depth knowledge and moral awareness they need to approach the collection and use of biospecimens with competence.

The idea of biobanking being taught in the medical curriculum also aligns with the existing paradigm of the competency-based curriculum, where the experience of research and moral responsibility in the practice are at the center of the scientific focus (Zohouri and Ghaderi, 2020). This information on the curriculum will assist in producing professionals who are competent in both scientific research and care of the patient. Lastly, adding instruction on biobanking to medical programs can improve medical research and education. This contributes to an academically motivated, scientific, and evidence-based healthcare regime.

New findings in the Middle East and Central Asia demonstrate similar decree patterns of moderate knowledge and high levels of altruistic motivation, such as the ones published in Iran (Domaradzki et al., 2025) and in Kazakhstan (Walkowiak et al., 2024), and earlier studies by Saudi indicated similar attitudes and concerns (Buhmeida et al., 2022). These appear to be congruent with the European and North American research findings of willingness to give data as being driven by a perceived social good but checked by privacy and data sharing issues. Putting the existing findings into this broader literature will help to align Saudi Arabian student attitudes with those of other nations.

### 5.3 Study’s implications

This study highlights the importance of biobanking in medical education and offers proof that it can be successfully incorporated into curricula. It will make students better understand essential concepts, ethical considerations, and processes of biospecimen donation.

Making biobanking issues and bioethics subjects common to curriculum orientations ensures that future practitioners are conversant with the research model. The results indicate that the change of the biobanking concepts will enhance the knowledge of students in regard to the ethics of donations by making them more informed and morally conscious. The situation is validated, as students have been very supportive of the effective use of learning modules that are engaging and informative in nature. The result justifies the increasing importance of biobanking to define medical research and aligns with the current reforms that are fostering research literacy and making graduates leaders in science in the present times.

The paper justifies the reasons why privacy and ethical issues should be appropriately dealt with. The trust needs to be developed to facilitate voluntary donation of biospecimens. Incorporating biobanking into education improves the general operation and efficacy of biobanks as well as student training.

To have a clear picture of the views of the future healthcare workers is the key aspect of ethical, effective biobanking. Any value of donation and concern should be made clear in communication plans, while insecurities should be addressed to eliminate the deterrents. This method promotes the culture of informed consent and ethics, which will establish a supportive culture.

The results indicate the impact that ethics have on the organizational culture of biobanks. Establishing trust will involve stringent privacy policies, understanding consent agreements, and moral norms. In biobanks, the donors must be made to feel secure, rewarded, and motivated by the social good they engage in. The study provides practical solutions to improving medical education and ethical operation of biobanks and raising more participative donors by instilling trust, transparency, and education.

### 5.4 Limitations

There are other restrictions that must be identified. Firstly, nearly one-third of the respondents were first/third-year students with minor experience with the ethics involved in research, which could have been one of the reasons behind the low level of knowledge and less confident attitudes. Second, a survey was sent to 457 students, but there is no ability to determine the response rate, and the effective sample might be lower than optimal. Third, the research was done in a single institution, and this curtails the generalization to the entire group of Saudi Arabian medical students. Fourth, the data were gathered online, which means that there is a likelihood of leaving out individuals who are less able to access the internet or those who are less interested in online surveys. Lastly, the research quantified hypothetical willingness to engage in biobanking, not necessarily a full manifestation of real-life behavior.

The study’s drawback of not including students from non-medical fields probably diminished the range of viewpoints on biobanking. Contributions from disciplines like public policy, ethics, and law are important because they can offer vital, different perspectives on the moral, legal, and societal ramifications of biobanking. Another limitation is associated with the self-reported features of the data. The respondents might have responded to questions as they saw it as the most wanted or socially acceptable, which can result in the existence of possible response bias. This can tamper with the validity of the results. This limitation can be addressed by future researchers through the inclusion of objective data, such as behavioral observations or ratings from other individuals, and self-reported data.

The study was also cross-sectional, implying the sampling of opinions at one moment in time. Even though it is a useful tool in figuring out the patterns, it cannot result in cause-and-effect conclusions. It also fails to capture how attitudes may be transformed over time. A longitudinal study would help in cashing in on the changes in perceptions during the academic period of the students and give an in-depth report on the changes in perceptions.

Despite being based on previous research and proving to be valid, the questionnaire’s structured format might have forced participants to give straightforward responses rather than allowing for more nuanced self-expression that might have exposed more complex attitudes. The set of questions provided in the survey may be insufficient to uncover advanced views held by the medical students and interns. The limitations of the study demonstrate that more comprehensive and heterogeneous studies are necessary. The integration of feedback, mixed research, and longitudinal design brings out enlightening results on attitudes to biobanking. These reflections suggest that more comprehensive and multi-institutional studies should be developed and that the designs require the ability to measure both professed intentions and actual participation behavior.

## 6 Conclusion and recommendations

### 6.1 Conclusion

The article provides the perspectives of medical students on the concept of biobanking at Alfaisal University. The data shows that although people have a moderate level of factual knowledge about biobanking, they strongly recognize its value in medical research. The benefits of society and the speed at which personalized therapies can evolve impress the students. Nevertheless, they are also afraid of personal information abuse, information leakage, or retaliation. This uncertainty displays the mistrust in the benefits and protection of biobanking. It is essential not just to point out the benefits. Ethical and prosperous biobanking in Saudi Arabia requires a two-pronged approach. First, biobanking education, including the technical, moral, and legal sides, has to be introduced into medical curricula to produce informed physician-advocates. Second, biobanking organizations must take keen initiative to put in place adequate measures that protect the rights of participants and their information privacy. Enhanced trust and knowledge in the medical community will change the positive attitudes to confident involvement and open up the potential of biobanking as a foundation of biomedical research in the future.

### 6.2 Recommendations

The first training modules in medical schools should be based on biobanking involving operational principles, social benefits, and above all, ethical standards of informed consent, anonymity, and privacy of data. These modules will seal gaps in the knowledge. Meanwhile, biobanking organizations should focus on high levels of transparency and successful communication to reassure the donors that their rights and data will not be abused or discriminated against. The creation of an atmosphere of trust and generosity is possible through the use of already existing altruistic incentives to stimulate early and continuous student involvement in the form of seminars and participation opportunities. To better understand the changing obstacles and enablers to biobanking participation, future research in Saudi Arabia should take a longitudinal approach and broaden its sample to include the general population.

## Data Availability

The raw data supporting the conclusions of this article will be made available by the authors, without undue reservation.
