# Supplementary material for: Biobanking as a research accelerator: the perspectives of medical students and interns at a saudi university
Source: Front Genet. 2026 Jan 7;16:1687927. doi: 10.3389/fgene.2025.1687927 (PMC12818786; doi:10.3389/fgene.2025.1687927)
Supplement: Supplementary file 1 [file Table1.docx]

**Supplementary Material**

Greetings,

We are a team of Master’s candidates from the College of Medicine at Alfaisal University, and we would like to invite you to participate in our study titled “Knowledge and attitudes of Al Faisal University Medical Students towards Biobanking as a Research Accelerator” Your participation in the survey below is voluntary; the responses will be kept confidential and used for research purposes only. Filling out the survey will not take more than 5 minutes.

*We highly appreciate your cooperation.*

*Thank you*

Participation in this study is voluntary, and you have the full right not to accept the questionnaire; choosing yes is proof of your informed consent to participate in this study.

☐ Yes

☐ No

**I. The first section of this questionnaire focused on demographics and medical background:**

1. **Age:**

☐ (Manual Entry)

1. **Gender:**

☐Male ☐Female

1. **Nationality:**

☐Saudi ☐Non-Saudi

1. **Current academic level at your College of Medicine:**

☐1^st^ year ☐2^nd^ year ☐3^rd^ year ☐4^th^ year ☐5^th^ year ☐Internship

1. **How would you rate your general health status?**

☐Excellent ☐Very Good ☐Good ☐Fair ☐Poor

1. **Has a doctor or any health professional ever diagnosed you with a chronic disease (e.g. heart disease, diabetes, stroke, cancer…etc.)?** ☐Yes ☐No

1. **Is there a history of inherited disease in your family?** ☐Yes ☐No

1. **Have you ever had a blood test?** ☐Yes ☐No

1. **Have you ever donated blood?** ☐Yes ☐No

1. **Have you ever had a tissue test (e.g., biopsy, buccal swab…. etc.)?** ☐Yes ☐No

1. **Have you ever donated tissue/an organ?** ☐Yes ☐No

1. **Have you ever been a participant in health-related research?** ☐Yes ☐No

**II. The second section of this questionnaire focused on your knowledge towards biobanking:**

1. **Have you ever heard of the term “biobank”?** ☐Yes ☐No

1. **What do you think the purpose of a “biobank” is?**

- Collect and store biospecimens for diagnostic and treatment purposes only
- Collect and store biospecimens for research purposes only
- Collect and store biospecimens for diagnostic, treatment, and biomedical research purposes
- Do not know

**15. According to modern biobanking, biospecimens mean:**

- Samples and/or biomolecules only
- Samples and/or biomolecules with annotated clinical data only
- Samples and/or biomolecules with annotated clinical, socioeconomic and lifestyle data 🞎 Annotated clinical, socioeconomic and lifestyle data only
- Do not know

**16. If yes, why? (You may select more than one answer)**

- it will nourish medical research, benefit society and future generations
- it will accelerate the curing of some instances through precision medicine 🞎 it will notify me about abnormal results

**17. If no or not sure, why? (You may select more than one answer)**

- Concern about the misuse of biospecimens in biomedical research
- Concern about confidentiality and privacy breaches
- Concern that biological or personal information may be used for discriminatory purposes 🞎 Fear of pain associated with biospecimen donations, such as injections and needle pricks 🞎 Religious reasons

1. **Do you think that biospecimen annotated data (i.e., related data) should be confidential and anonymous?**

☐Yes ☐No ☐Do not know

1. **Do you think donating a biospecimen to a biobank requires signing a consent form?**

☐Yes ☐No ☐Do not know

1. **Do you think that there is a standard operating procedure (SOPs) for biobanks to collect, process, store and release biospecimens?**

☐Yes ☐No ☐Do not know

**III. The third section of this questionnaire focused on the attitudes toward biomedical research in general:**

| **Do you agree or disagree with each of the following statements?** | **Strongly Agree** | **Agree** | **Neutral** | **Disagree** | **Strongly disagree** |
| --- | --- | --- | --- | --- | --- |
| 21. I have a positive view of biobanking in medical research |  |  |  |  |  |
| 22. I will donate a biospecimen (e.g., saliva/sputum, urine, blood, buccal swabs, toenails, hair, my own excess surgical tissue, a deceased family member’s organs or tissues, etc.) to a biobank to perform biomedical research |  |  |  |  |  |
| 23. Medical researchers are mainly motivated by personal gain |  |  |  |  |  |
| 24. Medical researchers can be trusted to protect the interests of people who take part in their research |  |  |  |  |  |
| 25. Medical researchers can influence others by volunteering for medical research |  |  |  |  |  |
| 26. Participating in medical research is generally safe |  |  |  |  |  |
| 27. Modern science poses more harm than good |  |  |  |  |  |
| 28. Society needs to devote more resources to medical research |  |  |  |  |  |
| 29. Medical research needs to be closely regulated in order to prevent harm to research participants |  |  |  |  |  |
| 30. Medical research will find cures for many major diseases during my lifetime |  |  |  |  |  |
